# Fine-Needle Aspiration Biopsy and Endoscopic Ultrasound for Pretreatment Pathological Diagnosis of Gastric Gastrointestinal Stromal Tumors

**DOI:** 10.1155/2012/139083

**Published:** 2012-09-26

**Authors:** Hiroaki Ito, Haruhiro Inoue, Shomei Ryozawa, Haruo Ikeda, Noriko Odaka, Nikolas Eleftheriadis, Roberta Maselli, Norimasa Sando, Satoshi Kimura, Shin-ei Kudo

**Affiliations:** ^1^Digestive Disease Center, Showa University Northern Yokohama Hospital, 35-1 Chigasakichuo, Tsuzuki-ku, Yokohama 224-8503, Japan; ^2^Central Clinical Laboratory and Department of Laboratory Medicine, Showa University Northern Yokohama Hospital, 35-1 Chigasakichuo, Tsuzuki-ku, Yokohama 224-8503, Japan

## Abstract

*Background*. Although it is possible to visualize gastrointestinal stromal tumors (GIST) of the stomach by endoscopy, their pretreatment histological diagnosis is often difficult. The aim of this study was to investigate predictors of accurate preoperative pathological diagnosis of gastric GIST. *Material and Methods*. We retrospectively studied patients with gastric GIST who had undergone pretreatment endoscopic biopsy and surgery, and examined their clinicopathological data. *Results*. Twenty-three patients were eligible. Thirty-four endoscopic biopsies (mean 2.6, range 1–8) were obtained. Preoperative pathological diagnoses of GIST were made in 18 patients. Precise diagnoses were made in 18 (52.9%) of the 34 biopsies. Endoscopic ultrasound (EUS) resulted in precise diagnoses in 11 (91.7%) of the 12 biopsy specimens. Fine-needle aspiration (FNA) biopsy resulted in precise diagnoses in 11 (84.6%) of the 13 biopsy specimens. The accuracy of pathological diagnosis by EUS-guided FNA biopsy was 100%. The procedure of EUS-guided FNA biopsy had no complications or recurrent disease. In a multivariate analysis, only EUS achieved a significantly superior rate of diagnosis (odds ratio, 11.884; 95% confidence interval, 1.204–289.230; *P* = 0.034). *Conclusion*. EUS-guided FNA biopsy is the most accurate for pretreatment pathological diagnosis of gastric GIST and for prevention of both of early complications and disease recurrence.

## 1. Background

Gastric submucosal tumors (SMTs), a rare disease, are often found incidentally during gastric surgery [1]. The commonest gastric SMT is gastrointestinal stromal tumor (GIST) [2]. GISTs are the commonest mesenchymal subepithelial tumors of the gastrointestinal tract. Although most small GISTs are benign tumors, risk classification for GIST by mitotic index, size and tumor site was provided [3]. 

Although biopsies generally provide a histological diagnosis and thus facilitate planning of the treatment strategy, it is sometimes difficult to make a pathological diagnosis of gastric SMTs because they are covered by normal gastric mucosa. In addition, biopsies of gastric GISTs may cause tumor rupture and dissemination [3]. Thus, gastric GISTs are often evaluated by computed tomography (CT) [4], magnetic resonance imaging (MRI) [5], and positron emission tomography (PET) [6].

Some new biopsy techniques [7, 8] for safe and effective diagnosis of gastric SMT have recently been developed. The usefulness of fine-needle aspiration (FNA) biopsy has also been reported [9–11]. The most powerful advantage of FNA biopsies is that physicians can extract specimens from beneath the mucosal layer. Furthermore, combination with endoscopic ultrasound (EUS) allows more precise assessment of the depth of the biopsy point. The disadvantages of FNA biopsy include its technical difficulty, the necessity for some exclusive instruments, and the occurrence of complications. GISTs often disseminate through the peritoneum. Accidental perforation of the gastric wall by a poorly performed FNA biopsy could cause iatrogenic peritoneal dissemination [3]. Accordingly, the treatment strategy for gastric GISTs is often planned based only on clinical findings without a pathological diagnosis. Because this is unsatisfactory, better diagnostic methods are under development. 

The aim of this retrospective study was to investigate the efficacy and safety of biopsies for gastric GIST. 

## 2. Patients and Methods

### 2.1. Patients

All patients who had undergone surgical resection and pathologically diagnosed gastric GIST after surgery, at the Digestive Disease Center, Showa University Northern Yokohama Hospital between April 2001 and March 2012 were retrospectively studied. 

The inclusion criteria were: (i) solitary submucosal tumor, (ii) pretreatment endoscopic biopsy performed, (iii) no prior treatment by endoscopic resection, surgery, chemotherapy, or radiation therapy. The exclusion criterion was synchronous malignancy.

The accuracy of the preoperative histological diagnoses and any complications of the pretreatment biopsies were assessed. Patient clinical records and pathology reports were reviewed to identify the clinical characteristics of the tumors as assessed by endoscopy and CT, biopsy techniques, and pathological diagnoses of the biopsy and surgical specimens.

The formalin-fixed biopsy and surgical specimens were prepared for diagnosis as follows. Hematoxylin and eosin, and immunohistochemical staining using antibodies of CD117 (Dako-Japan, Tokyo, Japan), CD34 (QBEnd10, Dako-Japan), alpha smooth muscle actin (*α*SMA) (1A4, Dako-Japan), desmin (D33, Dako-Japan) and S-100 (S-100, Dako-Japan) were performed. Tumors that stained positively for CD117 or CD34 were diagnosed as GISTs. Tumors that stained negatively for CD117 and CD34, and positively for *α*SMA or desmin, were diagnosed as leiomyomas. Tumors that stained negatively for CD117 and CD34, and positively for S-100, were diagnosed as schwannomas. Risk was graded according to the National Institutes of Health risk grading system [12]. The pathological diagnoses were made by agreement of two or more examiners, including at least one board certified pathologist. 

### 2.2. Statistical Analysis

The *χ* ^2^ test (Fisher's exact test and Pearson's test) was used for univariate analysis. Variables showing a univariate association (*P* < 0.10) were subjected to multivariate analysis. For multivariate analysis, a multiple logistic regression analysis yielding odds ratios and 95% confidence intervals was used to identify independent predictors that related accuracy of pathological diagnosis by biopsy. *P* values of less than 0.05 were considered to indicate statistical significance. Statistical analysis was performed using JMP Statistical Discovery 9.0.2 (SAS Institute, Cary, NC, USA).

The study was approved by the Institutional Review Board of the Showa University, Northern Yokohama Hospital (no. 1203-01). The research reported in this paper was in compliance with the Helsinki Declaration. This study was registered with the University Hospital Medical Information Network in Japan (no. UMIN000007428).

## 3. Results

**Figure 1:**
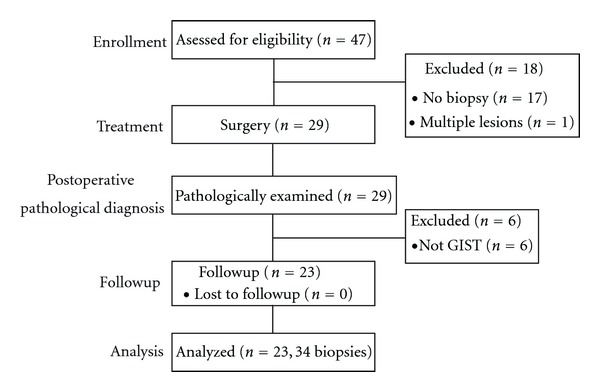
Enrollment of patients included in the study. Total 47 patients had undergone surgical resection for gastric submucosal tumors at the Digestive Disease Center, Showa University Northern Yokohama Hospital between April 2001 and March 2012 were retrospectively studied. The 23 patients pathologically diagnosed GIST after surgery were eligible.

Altogether, 23 patients (12 males and 11 females, mean age 60.0 years) were eligible (Figure 1). The median follow-up period was 20 months (range 1–85). The clinicopathological characteristics are summarized in Table 1. In the 23 patients, endoscopic biopsies were performed 34 times (mean 1.4, range 1–5). Before treatment, 18 (78.3%) and one (4.3%) patient were pathologically diagnosed as having GISTs and leiomyoma, respectively; no pathological diagnosis was made in the other four patients (17.4%). The postoperative pathological diagnoses of all 23 patients were GIST. Thirteen patients had a low GIST risk group, five intermediate, and five high (Table 2).

### 3.1. Statistical Analysis

The pathological diagnoses on biopsy and surgical specimens were the same for 18 (52.9%) of the 34 biopsies. Univariate analysis showed that agreement between pathological diagnoses on biopsy and surgical specimens was statistically significant for EUS and FNA biopsies (*P* = 0.001 and 0.005, resp.). Eleven of 12 (91.7%) EUS biopsy specimens and 11 of 13 (84.6%) FNA biopsy specimens were correctly diagnosed. Sex and age were not significantly associated with correct biopsy diagnoses. Clinical tumor size, using various cutoff lines including 30, 40 and 50 mm in diameter, was not significantly related to correct biopsy diagnoses. Presence or absence of ulceration did not correlate with accuracy of diagnosis. The times of endoscopy or biopsy were also not significant (Table 2). 

In a multivariate analysis, only EUS was a significant factor (odds ratio, 11.884; 95% confidence interval, 1.204–289.230; *P* = 0.034). Although this difference was not statistically significant, FNA biopsies were more accurate than forceps biopsies (odds ratio, 3.102; 95% confidence interval, 0.316–30.964; *P* = 0.312) (Table 3).

To assess the relationship between EUS and FNA biopsies, all 34 biopsies were categorized by the presence of EUS and FNAB as follows: EUS-guided FNA biopsy (EUS+/FNAB, 10 biopsies), forceps biopsy with EUS (EUS+/forceps, 2 biopsies), FNA biopsy without EUS (EUS−/FNAB, 3 biopsies), and forceps biopsy without EUS (EUS−/forceps, 19 biopsies). The accuracy of pathological diagnoses of biopsy specimens by EUS+/FNAB, EUS+/forceps, EUS−/FNAB, and EUS−/forceps was 100%, 50.0%, 33.3%, and 31.6%, respectively. There were significant differences between these four groups according to Pearson's test (*P* = 0.005). Comparison showed that the EUS+/FNAB group had significantly higher diagnostic accuracy than the EUS−/FNAB and EUS−/forceps groups (*P* = 0.038 and *P* = 0.0004, resp.). Although this difference was not statistically significant, the EUS+/FNAB group showed higher accuracy than the EUS+/forceps group (Figure 1). 

### 3.2. Patients' Clinical Courses

Of the 23 patients, two in whom forceps biopsies had been performed developed recurrent disease. One of these patient developed liver metastases 15 months after surgery, the other developed liver metastases and peritoneal dissemination 22 months after surgery. No patients in whom FNA biopsies had been performed had disease recurrences. 

## 4. Discussion

Gastrointestinal stromal tumors are the commonest mesenchymal subepithelial tumors of the gastrointestinal tract. They originate from the interstitial cells of Cajal. Thus, they are mainly located in the submucosal to muscular layer of the digestive tract and present as submucosal tumors. Because GISTs smaller than 2 cm are rarely associated with symptoms, small ones are usually detected incidentally during abdominal surgery or radiological examinations [13]. Small GISTs are considered benign, those of more than 50 mm have malignant potential and can metastasize to the liver and peritoneum [3]. In addition to size, the mitotic index is reportedly a significant risk factor for malignant potential [12]. Immunohistochemistry tests show that GISTs are positive for *KIT *(CD117; 95%), CD34 antigen (70%), SMA (30%–40%), desmin (<5%), and S-100 protein (<5%) [12].

The treatment strategies for neoplastic tumors are generally based on their histological characteristics. Of the gastric submucosal tumors, leiomyomas, neurilemmomas (schwannomas), and other benign tumors do not generally require any treatment; potentially malignant tumors such as GISTs, leiomyosarcomas, and carcinomas do require treatment because of the possibility that they will invade and metastasize. Because submucosal tumors are covered by normal mucosa, obtaining a specimen of tumor tissue by endoscopic biopsy is often difficult. Accordingly, various biopsy techniques have been developed [7, 10]. However, some authors do not recommend biopsy for diagnosis of GISTs because of the possibility of facilitating their dissemination [14]. 

Accordingly, diagnosis and treatment strategies for GIST have been mainly determined based on the findings of radiological investigations such as CT [4], MRI [5], and PET [6]. Though surgical tumor resection is recommended for localized GISTs [3], it is not so easy to distinguish them from benign submucosal tumors such as leiomyomas and schwannomas, for which surgical resection is not indicated. However, failure to resect significant gastric submucosal tumors for which no definite pathological diagnosis has been established is potentially dangerous, as malignant tumors could thus remain untreated. 

Endoscopic ultrasound-guided FNA biopsy is reportedly useful for diagnosing submucosal tumors [9–11]. The diagnostic accuracy of EUS-guided FNA biopsy for GISTs is 80–100% [9, 10]. On the other hand, the National Comprehensive Cancer Network (NCCN) advises that biopsy may not be necessary for easily resectable tumors. The NCCN also states that core needle biopsy (CNA) samples are preferred to EUS-guided FNA biopsies, because CNA samples can provide information about mitotic rate for assessment of risk [3].

In this study, univariate analysis showed that EUS and FNA biopsies were predictors for accurate pretreatment diagnosis of gastric GISTs. Multivariate analysis showed that EUS was the only significant factor. Although this difference was not statistically significant, FNA biopsies were more accurate than forceps biopsies. Tumor-related characteristics such as tumor size and presence of ulceration did not correlate significantly with accurate pretreatment diagnoses. These results suggest that EUS and FNA biopsy procedures are capable of obtaining adequate samples of tumor tissue for pretreatment pathological diagnosis regardless of the tumor characteristics. 

**Figure 2:**
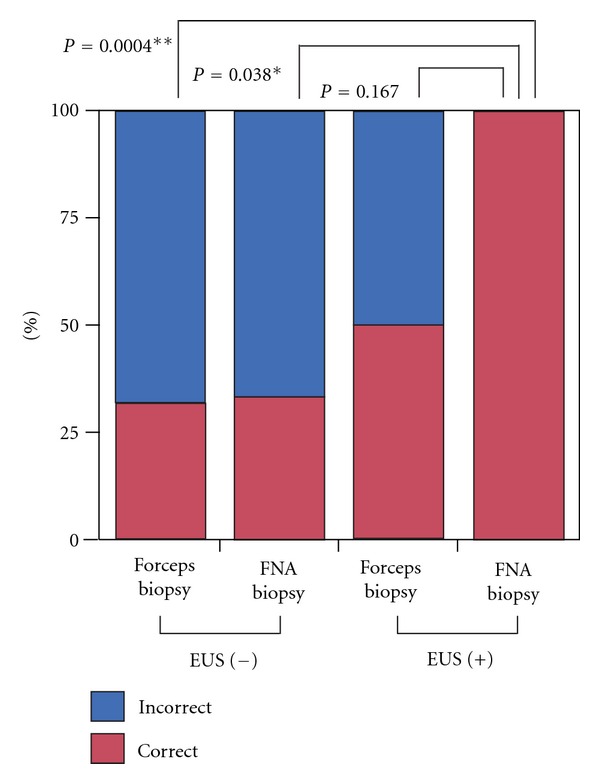
Consistency of pathological diagnosis between biopsy and surgical specimens. All 34 biopsies were categorized by presence of EUS and FNAB as EUS-guided FNA biopsy (EUS+/FNAB, 10 biopsies), forceps biopsy with EUS (EUS+/forceps, 2 biopsies), FNA biopsy without EUS (EUS−/FNAB, 3 biopsies), and forceps biopsy without EUS (EUS−/forceps, 19 biopsies). The EUS+/FNAB group had significantly better diagnostic accuracy than the EUS−/FNAB and EUS−/forceps groups (*P* = 0.038 and *P* = 0.0004). Although this difference was not statistically significant, the EUS+/FNAB group had better accuracy than the EUS+/forceps group.

We attempted to determine whether EUS or FNA biopsies are preferable for the diagnosis of GISTs. Of 13 FNA biopsies, 10 were performed using EUS (EUS-guided FNA biopsy) and three without EUS. All 10 EUS-guided FNA biopsy specimens were accurately diagnosed as GIST. In contrast, the accuracy of diagnostics samples obtained by forceps and FNA biopsies without using EUS was 31.6% and 33.3% (Figure 2). The diagnostic accuracy of specimens obtained by forceps biopsies using EUS was 50% also not so high (Figure 2). These results show that FNA biopsy is a necessary, but not independent, predictor of accuracy of diagnosis and that a combination of EUS and FNA biopsy (EUS-guided FNA biopsy) is the most effective means of achieving an accurate pathological diagnosis of a biopsy specimen.

We experienced no major complications in 34 biopsies, including FNA biopsies. Additionally, no patient who underwent FNA biopsy has developed disease recurrence.

Accordingly, we have concluded that EUS-guided FNA biopsy is useful for pretreatment pathological diagnosis of gastric GIST and prevention both of early complications and late idiopathic disease recurrence. We are now working on a study with a larger sample size to confirm the efficacy of EUS-guided FNA biopsy for diagnosis of submucosal tumors of the digestive tract. 

## 5. Conclusion

In this study, EUS-guided FNA biopsies provided extremely accurate pathological diagnoses and were associated with no major complications or disease recurrence. The diagnostic accuracy of FNA biopsies without EUS is not good. The results of this small sample size study suggest that EUS-guided FNA biopsy is safe and useful for obtaining an accurate pretreatment pathological diagnosis of gastric GISTs. 

## Figures and Tables

**Table 1:** Patients' clinicopathological characteristics (*n* = 23).

Variables	Number of subjects
Sex	
Male	12
Female	11
Age (years, [mean, range])	60.0, 26–92
Main tumor site	
Upper third of stomach	16
Middle third of stomach	6
Lower third of stomach	1
Clinically determined tumor diameter (mm, [mean, range])	36.6, 18–60
Ulceration	
Present	12
Absent	11
Number of endoscopies (mean, range)	2.7, 1–8
Endoscopic ultrasound	
Yes	15
No	8
Number of biopsies/patient (mean, range)	1.4, 1–5
Fine-needle aspiration biopsy	
Yes	12
No	11
Preoperative pathological diagnosis	
Gastrointestinal stromal tumor	18
Leiomyoma	1
Normal gastric mucosa	4
Pathologically determined tumor diameter (mm, [mean, range])	45.4, 16–110
Gastrectomy	
Partial	20
Proximal	2
Distal	1
GIST risk group	
Low	13
Intermediate	5
High	5

**Table 2:** Univariate analysis (34 biopsies).

Variables	Accuracy of pathological diagnosis (correct/total)	*P* value
Sex		0.510
Male	9/15 (60.0%)	
Female	9/19 (47.3%)	
Age (years)		0.510
≤60	9/15 (60.0%)	
>60	9/19 (47.3%)	
Clinically determined tumor diameter (mm)		1.000
≤30	7/14 (50.0%)	
>30	11/20 (55.0%)	
Ulceration		0.327
Present	10/16 (62.5%)	
Absent	8/18 (44.4%)	
Number of endoscopy		1.000
First	8/16 (50.0%)	
Second or later	10/18 (55.6%)	
Endoscopic ultrasound		0.001**
Yes	11/12 (91.7%)	
No	7/22 (31.8%)	
Number of biopsy		0.274
First	14/23 (60.9%)	
Second or later	4/11 (36.4%)	
Biopsy procedure		0.005**
Normal	7/21 (33.3%)	
Fine-needle aspiration biopsy	11/13 (84.6%)	

**P < 0.01.

**Table 3:** Multivariate analysis.

Variable	Odds ratio	95% confidence interval	*P* value
Endoscopic ultrasound			
No	1.000	1.204–289.230	0.034*
Yes	11.884		
Biopsy procedure			
Forceps biopsy	1.000	0.316–30.964	0.312
Fine-needle aspiration biopsy	3.102		

*P < 0.05.

## Abbreviations

*α*SMA: Alpha smooth muscle actin.
CD: Cluster of differentiation.
CT: Computed tomography.
EUS: Endoscopic ultrasound.
FNA: Fine-needle aspiration.
FNAB: Fine-needle aspiration biopsy.
MRI: Magnetic resonance imaging.
PET: Positron emission tomography.
SMA: Smooth muscle actin.
SMT: Submucosal tumor.
